# Breaking the silence: how shedding light on the bone-skipper fly *Thyreophora cynophila* (Diptera: Piophilidae) demonstrated it still has a large distribution area in the Pyrenees mountains, France

**DOI:** 10.3897/BDJ.8.e54868

**Published:** 2020-09-16

**Authors:** Frédéric Azémar, Frédéric Cazaban, Laurent Pelozuelo

**Affiliations:** 1 Laboratoire d’Ecologie fonctionnelle et Environnement, UMR 5245, CNRS-INPT-UPS, 118 Route de Narbonne, 31062 Toulouse Cedex 09, France Laboratoire d’Ecologie fonctionnelle et Environnement, UMR 5245, CNRS-INPT-UPS, 118 Route de Narbonne 31062 Toulouse Cedex 09 France; 2 Unaffiliated, 30, rue Louis Jouvet, Tarnos, France Unaffiliated, 30, rue Louis Jouvet Tarnos France; 3 Office pour les Insectes et leur Environnement de Midi-Pyrénées, Muséum d'Histoire Naturelle, 2 place Philadelphe Thomas, 81600 Gaillac, France Office pour les Insectes et leur Environnement de Midi-Pyrénées, Muséum d'Histoire Naturelle, 2 place Philadelphe Thomas 81600 Gaillac France

**Keywords:** Lazarus species, lost species, insect extinction, silent extinction, quiet extinction, neglected majority, Pyrénées.

## Abstract

**Background:**

*Thyreophora cynophila* (Panzer, 1798) is an iconic species of the European entomofauna. This winter-flying necrophagous fly was considered long extinct in Europe, before being discovered in Spain in 2010 and re-discovered in France in 2020, with a unique locality in Saint-Paul-de-Jarrat (Ariège, southern France).

**New information:**

After bringing this species to the attention of people that are active in nature during the winter, including hunters, skilled naturalists, nature lovers and professional naturalists, we gathered seven new occurrence data for this species at six locations on the French flanks of the Pyrenees mountains. Those data considerably extend the known distribution of the species in Europe and allows mapping the first approximate extent of occurrence for this species in France.

## Introduction

Everyone is aware about the ongoing collapse of vertebrate populations (World Wildlife Fund 2016), from charismatic species, such as elephants, rhinoceros, tigers and apes, amongst others, to formerly common birds (Bird Life International 2020). We even give names to the last known specimen of some vertebrate species: Martha, the last passenger pigeon, Lonesome George, the last Pinta island tortoise, Sudan, the last northern white rhinoceros and Celia the last Pyrenean ibex. By contrast, most of us pay little attention to insects. Along with other invertebrates, they are the “little things that run the world” (Wilson 1987) and the “neglected majority” (Dunn 2005), which are being driven to “silent” or “quiet” extinction (Eisenhauer et al. 2019). Indeed, despite recent highlights on their drastic decline (Vogel 2017, Hallmann et al. 2017, Sánchez-Bayo and Wyckhuys 2019), very few people, even amongst entomologists, would be able to name any of the sixty-two insect species that are already extinct (IUCN 2019). Yet, awareness is a key issue to improve the knowledge of insect species and their conservation. We report here how shedding light on the iconic bone-skipper fly *Thyreophora cynophila* (Panzer, 1798) rapidly helped to map the approximate extent of occurrence of this species along the Pyrenees mountains in France. *Thyreophora cynophila* has an atypical phenology with the adult stage flying from November to March. It depends on large mammal carcasses in late stages of decay as a food resource and larvae are supposed to feed specifically on the marrow of large bones (Martín-Vega et al. 2010). *Thyreophora cynophila* original distribution used to include Germany, Austria, Algeria and France (Martín-Vega et al. 2010), but the species was long considered as extinct due both to the hygienic rules imposed on livestock carcass destruction and the regression of the wild ungulates megafauna (Fontaine et al. 2007). Menier 2003 even speculated that *T. cynophila* had become extinct because of the extirpation of the wolves, explaining that wolves used to provide carcasses with crushed bones, thus offering the fly access to bone marrow for their larvae. *Thyreophora cynophila* was rediscovered recently in Spain (Martín-Vega et al. 2010, Carles-Tolrá et al. 2010) and France, in Saint-Paul-de-Jarrat, Ariège Department (Léal et al. 2020). Both its phenology and its feeding habits are putative reasons why *T. cynophila* — like other Thyreophorine flies, *Centrophlebomyia furcata* (Fabricius, 1794) and *Thyreophora antropophaga* Robineau-Desvoidy, 1830 — has gone unnoticed for such a long time, in addition to the poor attention addressed to small insects. However, combined with the recognisable and eye-catching habitus of *T. cynophila* — a bright orange head with a shiny dark-blue body — those biological traits are useful to shed light on the species. During the winter of 2019, we invited people to report sightings of an orange-headed fly on winter carcasses and we gathered the reported sightings.

## Materials and methods

**Observers alert**

Hunters in the Region Occitanie, who are all members of the Regional Association “fédération des chasseurs d’Occitanie”, were invited on 25 and 26 November 2019 to look out for the eye-catching habitus of *T. cynophila* and to report putative sightings to L. Pelozuelo. Three media types were used: a tweet from “@frcoccitanie”, a post on their Facebook page (https://www.facebook.com/FedeChasseursOccitanie) and an actuality page on their website (https://www.chasse-nature-occitanie.fr/biodiversite-et-observatoire/actualites/a15342/chasseurs,-observateurs-par-nature).

Skilled naturalists and simple nature observers connecting to “Faune-Occitanie” (https://www.faune-occitanie.org) were alerted through an “actuality page” posted on 26 November 2019. “Faune Occitanie” is an internet portal managed by a consortium of more than 15 naturalist associations. It is dedicated to the compilation of occurrence data in animals. The equivalent websites for the national level “Faune France” (https://www.faune-france.org) and the Aquitaine Region “Faune Aquitaine“ (https://www.faune-aquitaine.org) also relayed the information, on 13 and 20 December 2019, respectively. Agents of the Pyrenees National Park were also alerted through two e-mails on 19 February 2019 and 18 November 2019.

## Data resources

**Figure 1.**
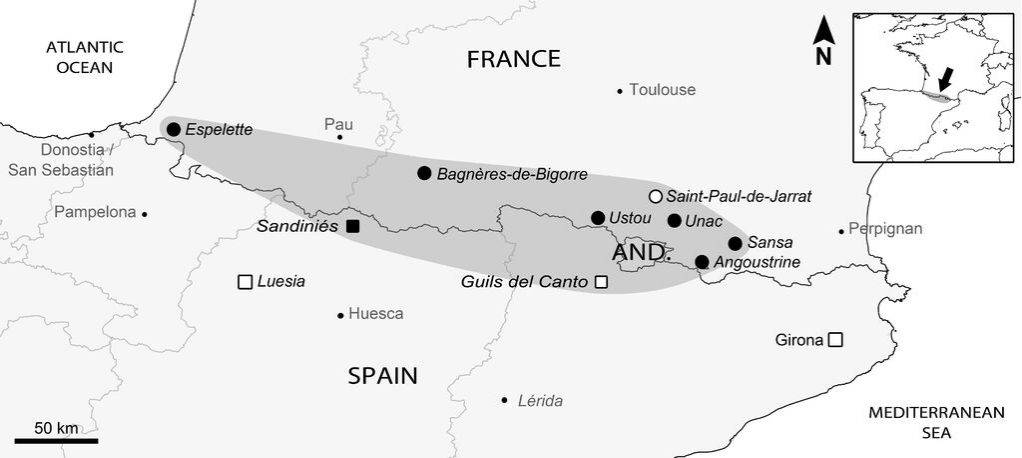
The extent of occurrence of the bone-skipper fly *Thyreophora cynophila* (Diptera: Piophilidae) in the Pyrenees mountain range. Data gathered during this study in winter 2019 (black circle). Observations in the Spanish Pyrenees (black square). Data obtained by Léal et al. 2020 (white circle). Closest data previously published for Spain (white sqare): Lérida, Carles-Tolrá 2013; Girona, picture by Juan Carlos Santiago (Biodiversidad virtual 2013) and Luesia, Carles-Tolrá et al. 2011.

A total of seven observations, supported by pictures of at least one adult *T. cynophila* individual, were reported from six localities (Table 1 ; Fig. 1) by seven different observers or groups of observers. Out of those seven observations, one was produced by a sheep breeder, one by agents of the French Office for Biodiversity and five were produced by people with naturalist skills. Another observation was obtained in Sandiniés (Spain, Province of Huesca), on the southern flanks of the Pyrenees. Due to the very characteristic habitus of *T. cynophila*, there was no need to capture or examine the flies further.

## Taxon treatments

### Thyreophora cynophila

(Panzer, 1798)

E8EE99E8-CFDC-5AFC-B846-16863F363A30

#### Materials

- **Type status:** Other material. **Occurrence:** individualCount: ≥ 20; lifeStage: adult; behavior: On deer carcass, Cervus elaphus; **Taxon:** scientificName: Thyreophora cynophila; acceptedNameUsage: Mouche gypaète, Mouche à tête orange, Thyréophore cynophile; order: Diptera; family: Piophilidae; genus: Thyreophora; specificEpithet: cynophila; scientificNameAuthorship: (Panzer, 1798); **Location:** continent: Europe; country: France; stateProvince: Occitanie; county: Pyrénées-Orientales; municipality: Angoustrine; verbatimElevation: 1476 m; verbatimCoordinates: 1°58'0.95" E 42°29'25.93" N; verbatimCoordinateSystem: decimal degrees; decimalLatitude: 42.490536; decimalLongitude: 1.966931; **Identification:** identifiedBy: L.Vallverdu; **Event:** samplingProtocol: Opportunistic sight
- **Type status:** Other material. **Occurrence:** individualCount: 1; lifeStage: adult; behavior: On semi-fera basque poney carcass, Equus caballus; **Taxon:** scientificName: Thyreophora cynophila; acceptedNameUsage: Mouche gypaète, Mouche à tête orange, Thyréophore cynophile; order: Diptera; family: Piophilidae; genus: Thyreophora; specificEpithet: cynophila; scientificNameAuthorship: (Panzer, 1798); **Location:** continent: Europe; country: France; stateProvince: Nouvelle-Aquitaine; county: Pyrénées-Atlantiques; municipality: Espelette; verbatimElevation: 482 m; verbatimCoordinates: 1°28'15.074" W 43°18'28.33" N; verbatimCoordinateSystem: decimal degrees; decimalLatitude: 43.307868; decimalLongitude: -1.470854; **Identification:** identifiedBy: F. Cazaban; **Event:** samplingProtocol: Opportunistic sight
- **Type status:** Other material. **Occurrence:** individualCount: ≥ 4; lifeStage: adult; behavior: On sheep carcass, Ovis aries; **Taxon:** scientificName: Thyreophora cynophila; acceptedNameUsage: Mouche gypaète, Mouche à tête orange, Thyréophore cynophile; order: Diptera; family: Piophilidae; genus: Thyreophora; specificEpithet: cynophila; scientificNameAuthorship: (Panzer, 1798); **Location:** continent: Europe; country: France; stateProvince: Occitanie; county: Aričge; municipality: Unac; verbatimElevation: 616 m; verbatimCoordinates: 1°46'23,31" E 42°45'19,20" N; verbatimCoordinateSystem: decimal degrees; decimalLatitude: 42.7553333; decimalLongitude: 1.773142; **Identification:** identifiedBy: P. Guiton; **Event:** samplingProtocol: Opportunistic sight
- **Type status:** Other material. **Occurrence:** individualCount: ≥ 8; lifeStage: adult; behavior: On doe carcass, *Cervus elaphus*; **Taxon:** scientificName: Thyreophora cynophila; acceptedNameUsage: Mouche gypaète, Mouche à tête orange, Thyréophore cynophile; order: Diptera; family: Piophilidae; genus: Thyreophora; specificEpithet: cynophila; scientificNameAuthorship: (Panzer, 1798); **Location:** continent: Europe; country: France; stateProvince: Occitanie; county: Pyrénées-Orientales; municipality: Angoustrine; verbatimElevation: 1885 m; verbatimCoordinates: 1°57'46" E 42°31'17 N; verbatimCoordinateSystem: decimal degrees; decimalLatitude: 42.516667; decimalLongitude: 1.95; **Identification:** identifiedBy: F.Caminade; **Event:** samplingProtocol: Opportunistic sight
- **Type status:** Other material. **Occurrence:** individualCount: 4; lifeStage: adult; behavior: On sheep carcass, *Ovis aries*; **Taxon:** scientificName: Thyreophora cynophila; acceptedNameUsage: Mouche gypaète, Mouche à tête orange, Thyréophore cynophile; order: Diptera; family: Piophilidae; genus: Thyreophora; specificEpithet: cynophila; scientificNameAuthorship: (Panzer, 1798); **Location:** continent: Europe; country: France; stateProvince: Occitanie; county: Aričge; municipality: Ustou; verbatimElevation: 873 m; verbatimCoordinates: 1°16'20,89" E 42°48'27,17"N; verbatimCoordinateSystem: decimal degrees; decimalLatitude: 42.807546; decimalLongitude: 1.27247; **Identification:** identifiedBy: X. Rozec, A. Pialot, L. Coutu; **Event:** samplingProtocol: Opportunistic sight
- **Type status:** Other material. **Occurrence:** individualCount: 1; lifeStage: adult; behavior: Dead. On Pyrenean chamois carcass, *Rupicapra pyrenaica*; **Taxon:** scientificName: Thyreophora cynophila; acceptedNameUsage: Mouche gypaète, Mouche à tête orange, Thyréophore cynophile; order: Diptera; family: Piophilidae; genus: Thyreophora; specificEpithet: cynophila; scientificNameAuthorship: (Panzer, 1798); **Location:** continent: Europe; country: France; stateProvince: Occitanie; county: Hautes-Pyrénées; municipality: Bagnères-de-Bigorre; verbatimElevation: 1965 m; verbatimCoordinates: 0°12'5,59" E 42°53'56,01" N; verbatimCoordinateSystem: decimal degrees; decimalLatitude: 42.898891; decimalLongitude: 0.201553; **Identification:** identifiedBy: M. and O. Taburet, M. Marlas; **Event:** samplingProtocol: Opportunistic sight
- **Type status:** Other material. **Occurrence:** individualCount: ≥ 3; lifeStage: adult; behavior: On Mediterranean wild sheep, *Ovis gmelini musimon* x *Ovis* sp.; **Taxon:** scientificName: Thyreophora cynophila; acceptedNameUsage: Mouche gypaète, Mouche à tête orange, Thyréophore cynophile; order: Diptera; family: Piophilidae; genus: Thyreophora; specificEpithet: cynophila; scientificNameAuthorship: (Panzer, 1798); **Location:** continent: Europe; country: France; stateProvince: Occitanie; county: Pyrénées-Orientales; municipality: Sansa; **Identification:** identifiedBy: A. Chapuis, L. Gayral; **Event:** samplingProtocol: Opportunistic sight

#### Description

**Figure 2.**
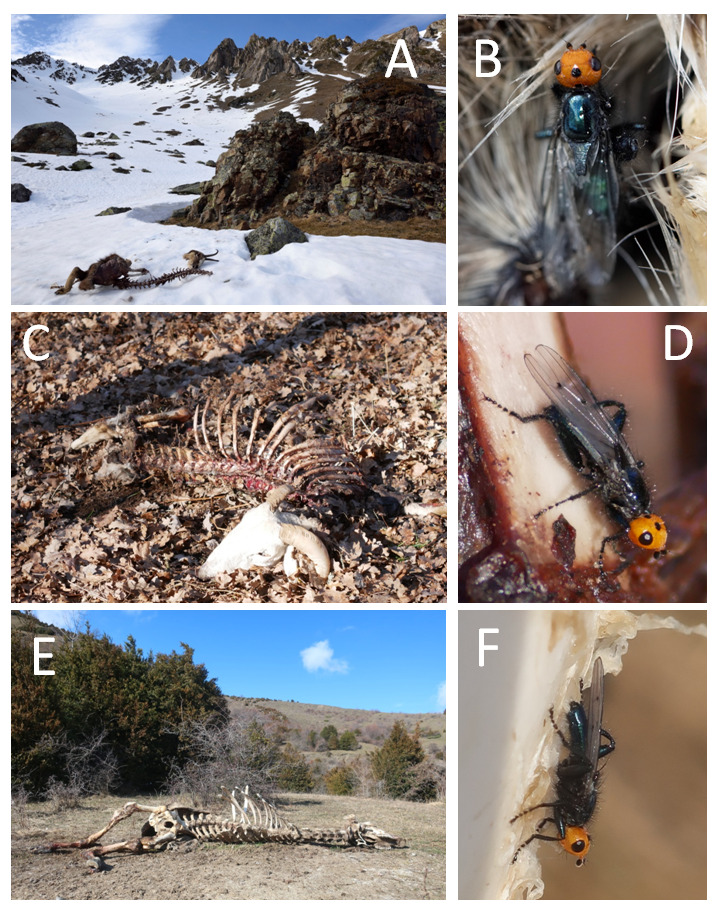
*Thyreophora cynophila* and its habitat. (A) & (B): Pyrenean chamois carcass and dorsal view of a *T. cynophila* individual found dead, probably due to low nocturnal temperature in Bagnères-de-Bigorre (Hautes-Pyrénées, France). Picture by Martin Taburet. (C) & (D) Sheep carcass and *T. cynophila* observed in Unac (Ariège, France). Picture by Pierre Guiton. (E) & (F). Cow carcass and *T. cynophila* observed in Sandiniés (Huesca, Spain). Picture by Laurent Pelozuelo.

*Thyreophora cynophila* is a mid-size fly (around 1 cm long) with a shiny black or dark-blue body and legs (Fig. 2). Its orange head is its most characteristic and eye-catching morphological trait. *Thyreophora cynophila* also has a long scutellum prolonged by two long hairs (Fig. 2B and D), a trait shared with other Piophilid flies, such as *Centrophlebomyia* sp. (Mei et al. 2013). Males have enlarged hind-legs.

## Discussion

Our seven observations are distributed along almost all of the Pyrenees mountain range, from Espelette, as the most western point, to Sansa approximately 300 km away to the east (Fig. 1), with Bagnères-de-Bigorre in the middle. The two sightings in Angoustrine and the sightings in Sansa, Unac and Ustou form a “cluster” close to Saint-Paul-de-Jarrat, where the species was re-discovered (Léal et al. 2020). Those localities are approximately 20 to 70 km away from each other (distances as the crow flies). No conclusion can be based on this pattern that may reflect a higher density of *T. cynophila* on the eastern side of the Pyrenees, but could also simply be the result of a biased sampling effort. Indeed, people close to the initial site of rediscovery may have felt more concerned and thus more prone to search for the fly.

Our observation in Sandiniés is the first observation in this northern Spanish province, on the southern flank of the Pyrenees. Based on our dataset and one previous observation in the south-eastern Pyrenees, in Guils del Cantó (Spain, Lérida Province, Carles-Tolrá 2013), the actual extent of occurrence in the Pyrenees mountain range can be mapped for the first time. It is approximately 14,000 km² in extent and rises between altitudes of 482 to 1,965 m. This cluster of observations is approximately 65 km away from the sighting in Luesia (Zaragoza Province, Carles-Tolrá et al. 2011, Carles-Tolrá 2011), 100 km away from a sighting in Girona (Biodiversidad virtual 2013), 170 km away from observations in Soria Province (Carles-Tolrá 2011, Carles-Tolrá et al. 2014) and 200 km away from a cluster of observations in La Rioja Province (Zaldívar Ezquerro et al. 2011).

*Thyreophora cynophila* could be found on both domestic and wild ungulate carcasses, which is consistent with previous reports in Spain (Morales et al. 2016) . This gives hope that the fly would benefit from an alternative resource in the case of sheep, cow and horse breeding decline for economic reasons. It also suggests that the species could take advandage of the effort dedicated to supplementary feeding programmes for vultures species, as the so-called "vulture restaurants" can host this fly (Martín-Vega and Baz 2011). However, we did not find *T. cynophila* on vulture restaurants during preliminary prospections in the eastern Pyrenees (Azemar & Pelozuelo, unpublished data). Moreover, sanitary rules for the management of vulture restaurants in France are not compatible with the support of *T. cynophila* as carcass remains, including bones, must be removed within seven days (République Française 1998). Even if we verified that this seven-days delay is poorly respected, vulture restaurant managers collect and incinerate the remaining bones at least once a year and this may negatively impact *T. cynophila*. A modification of the sanitary rules to allow a longer time for bones remaining in the vulture restaurants would thus probably be beneficial for *T. cynophila*. Considering its former presence in the surroundings of Paris, Manheim or Algier (Martín-Vega et al. 2010) and its actual large distribution in Spain (Morales et al. 2016), *T. cynophila* probably depends on large carcasses availability rather than on habitat type (i.e vegetation or soil), as do the endangered carrion beetle *Nicrophorus americanus* Olivier, 1790 ([Bibr B5997240][Bibr B5997540]). Thus, if conservation measures were necessary, they probably should focus on the availability of large carcasses. For the moment, the ongoing "re-wilding" of the Pyrenees with stable or increasing populations of six wild ungulates species (Ibex, *Capra pyrenaica pyrenaica*; Pyrenean chamois, *Rupicapra pyrenaica pyrenaica*; deer, *Cervus elaphus*; roe deer, *Capreolus capreolus*; mediterranean wild sheep *Ovis gmelini musimon x Ovis* sp. and wild boar, *Sus scrofa* - and the increasing population of brown bear *Ursus arctos* (Sentilles et al. 2020) plus the arrival of the wolf *Canis lupus)* should allow *T. cynophila* to escape extinction.

Such a picture is quite positive as it demonstrates that the “absence” of *T. cynophila* in France was due to a lack of attention. It lets us expect new sightings in the future, particularly in other mountain ranges where sheep, cow and horse breeding are still present and co-exist with populations of wild ungulates (Alps, Massif Central). Large territories where agro-pastoralism is still important enough to support vulture populations, like the “Great Causses” (close to Millau, Aveyron Department), may also host *T. cynophila* populations. *T. cynophila* is definitively a lazarus species now "back from the dead" (Martín-Vega et al. 2010, Léal et al. 2020) like *Centrophlebomyia furcata* that was re-discovered in Spain (Gómez-Gómez et al. 2008). Nevertheless, the conservation status of *T. cynophila* cannot be evaluated due to the lack of data. Its area of occurence has probably been severly reduced in France and it is hardly imaginable that a contemporary dipterist would find it in the surroundings of Paris as Robineau-Desvoidy did in 1836 (Robineau-Desvoidy 1841). However, recent observations in Spain and France should encourage prospection targeting this species in other parts of France, in Germany and Austria where it has been previously observed, but also in other countries of central Europe (for example, in the primeval Białowieża forest in Poland) and in Algeria.

We expect that increased awareness and new tools such as websites and smartphone applications dedicated to report and gather occurrence data (Inaturalist, Naturalist, Faune-France) will increase our knowledge of the geographic distribution of this iconic species and related taxa, such as *Centrophlebomyia* sp. in France and Europe.

## Supplementary Material

XML Treatment for Thyreophora cynophila

## Figures and Tables

**Table 1.** Occurrence data of the bone-skipper fly *Thyreophora cynophila* (Diptera: Piophilidae) in France gathered during winter 2019/2020. Department: 09: Ariège. 64: Pyrénées-Atlantiques. 65: Hautes-Pyrénées. 66: Pyrénées-Orientales.

**Locality (Department)**	**Coordinates (decimal degrees)**	**Altitude (m)**	**Date**	**Number**	**Carcass**	**Observer**
Angoustrine, (66)	42.490536, 1.966931	1476 m	23/11/2019	≥ 20	Deer, *Cervus elaphus*	L. Vallverdu
Espelette, (64)	43.307868, -1.470854	482 m	02/01/2020	1	Semi-feral Basque poney, *Equus caballus*	F. Cazaban
Unac, (09)	42.7553333, 1.773142	616 m	09/01/2020	≥ 4	Sheep, *Ovis aries*	P. Guiton^1^
Angoustrine, (66)	42.516667, 1.950000	1885 m	01/02/2020	≥ 8	Doe, *Cervus elaphus*	F. Caminade
Ustou, (09)	42.807546, 1.272470	873 m	02/02/2020	4	Sheep, *Ovis aries*	X. Rozec, A. Pialot, L. Coutu
Bagnères-de-Bigorre (65)	42.898891, 0.201553	1965 m	10/02/2020	1	Pyrenean chamois, *Rupicapra pyrenaica*	M. and O. Taburet, M. Marlas
Sansa, (66)	unknown	unknown	29/02/2020	≥ 3	Mediterranean wild sheep, *Ovis gmelini musimon x Ovis* sp.	A. Chapuis, L. Gayral^2^

^1^ Information transmitted by J.Aspirot (Observatoire de la Montagne).

^2^ Information transmitted by V. Lacaze (Association des Naturalistes de l’Ariège).
